# Professional Quality of Life, Team Cohesion, Organizational Commitment, and Intent to Leave Among Nursing Personnel: A Cross‐Sectional Study

**DOI:** 10.1155/jonm/9984390

**Published:** 2026-09-30

**Authors:** Daniel R. Kelly, Kristen Leighton, Rhoda A. Owens, Jana Zwilling, David Schmitz

**Affiliations:** ^1^ Master of Healthcare Administration Program York University York Nebraska USA yorku.ca; ^2^ Center for Rural Health University of North Dakota School of Medicine and Health Sciences Grand Forks North Dakota USA und.edu; ^3^ Department of Nursing University of North Dakota College of Nursing and Professional Disciplines Grand Forks North Dakota USA; ^4^ Department of Family and Community Medicine University of North Dakota School of Medicine and Health Sciences Grand Forks North Dakota USA und.edu

**Keywords:** burnout, nursing staff, occupational stress, personnel loyalty, personnel turnover, professional

## Abstract

**Aims:**

To examine whether professional quality of life, team cohesion, and organizational commitment were independently associated with intent to leave among nursing personnel.

**Background:**

Prior research links professional quality of life with turnover intention, but less is known about joint associations with team cohesion and multidimensional organizational commitment in nursing workforces.

**Methods:**

A cross‐sectional electronic survey of 633 nursing personnel in North Dakota used validated measures. Pearson correlations, multiple regression, and post hoc nested regression were conducted.

**Results:**

The full model explained 47% of intent‐to‐leave variance. Burnout was positively associated with intent to leave (*β* = 0.40, *p* < 0.001), whereas affective commitment (*β* = −0.41, *p* < 0.001) and continuance commitment (*β* = −0.10, *p* = 0.003) were inversely associated. Secondary traumatic stress (STS) was positively correlated with intent to leave (*r* = 0.16) but negatively associated after adjustment (*β* = −0.11, *p* = 0.005); its coefficient reversed when burnout entered.

**Conclusions:**

Intent to leave was associated with occupational strain and organizational attachment. The STS reversal is consistent with statistical suppression and should not be interpreted as protective.

**Implications for Nursing Management:**

Findings identify burnout and affective commitment as priorities for prospective strategy testing. Longitudinal research should determine whether modifying these domains reduces intent to leave or turnover.

## 1. Introduction

Nurse turnover disrupts continuity of care and imposes substantial recruitment, onboarding, and training costs on healthcare organizations. Rural and underserved organizations face particular retention challenges because staffing losses may be difficult to replace and can affect access to care. Burnout and secondary traumatic stress (STS) are consistently associated with poorer nurse well‐being and turnover‐related outcomes, whereas supportive work environments and organizational attachment can promote retention [1–3].

Turnover is especially consequential in rural and underserved settings, where smaller labor pools can make vacancies difficult to fill and increase workload pressure on remaining staff. Retention is therefore both a workforce objective and an operational priority for healthcare leaders [1].

Intent to leave is an important precursor to actual turnover because it captures employees’ consideration of voluntary separation before departure occurs. It is influenced by multiple individual and organizational conditions rather than a single determinant. In nursing, occupational stress, perceived support, professional opportunities, leadership practices, and attachment to the employing organization may interact to shape whether nurses envision remaining in or leaving their current positions. Examining these factors together is particularly useful in rural nursing workforces, where organizational options and community ties may differ from those in larger urban labor markets [4, 5].

Professional Quality of Life (ProQOL) encompasses compassion satisfaction and compassion fatigue; the latter includes burnout and STS [6]. Burnout reflects chronic occupational exhaustion and related negative responses to work, while STS reflects distress associated with indirect exposure to others’ trauma. Both constructs are relevant to turnover intention among nurses [7, 8].

Organizational commitment provides an organizational‐domain perspective on retention. Meyer and Allen’s [9] three‐component model distinguishes affective commitment (emotional attachment), continuance commitment (perceived costs of leaving), and normative commitment (a perceived obligation to remain) [9, 10]. Greater organizational commitment is generally associated with lower turnover intention among nurses [11, 12].

Team cohesion adds a relational dimension by reflecting shared goals, trust, collaboration, and constructive problem‐solving within work groups [13, 14]. Prior work suggests that cohesion and organizational commitment may protect nurse well‐being, although their independent contribution to intent to leave remains less clear when occupational stress and commitment are considered simultaneously [15, 16].

Recent studies have linked professional quality of life domains with turnover‐related outcomes in specialized nursing samples [17–20]. The contribution of the present study is therefore not an isolated professional quality‐of‐life–turnover association but the simultaneous estimation of the three professional quality‐of‐life domains, two team‐cohesion dimensions, and three organizational‐commitment dimensions in a statewide, mixed‐role nursing workforce with substantial rural representation.

### 1.1. Theoretical Rationale and Study Objectives

The study integrates constructs spanning occupational/individual, relational/team, and organizational domains. Professional quality of life reflects professional fulfillment and occupational strain; team cohesion reflects respondents’ perceptions of interpersonal resources within the work group; and organizational commitment reflects respondents’ attachment to the employer. All focal variables were measured at the individual respondent level; the domain labels describe construct content rather than levels of analysis. No team or organization‐level aggregation was performed. Examining these domains simultaneously allows assessment of their unique associations with intent to leave [5, 6, 9, 21].

The study objectives were to (1) describe professional quality of life, team cohesion, organizational commitment, and intent to leave among nursing personnel in North Dakota; (2) examine bivariate relationships among these variables; and (3) determine whether professional quality of life, organizational commitment, and team cohesion were independently associated with intent to leave.

## 2. Methods

### 2.1. Design, Setting, and Recruitment

A cross‐sectional electronic survey was conducted among nursing personnel employed in North Dakota healthcare settings between October 2024 and March 2025. For this study, “nursing personnel” refers to respondents employed in nursing‐related roles: registered nurses, licensed practical/vocational nurses, advanced practice nurses, certified nursing assistants, and other nursing‐related roles captured by the survey. Participants were eligible if they were 18 years of age or older and employed in a nursing‐related role in a North Dakota healthcare setting. Reporting was guided by the Strengthening the Reporting of Observational Studies in Epidemiology (STROBE) recommendations for cross‐sectional studies [22]. Convenience sampling was used. Recruitment announcements were distributed through statewide hospitals; long‐term care, rural health, and professional networks; electronic newsletters; organizational mailing lists; and social media. Because recruitment relied on open electronic dissemination through multiple channels, the number of individuals who received or viewed an invitation could not be established; therefore, a conventional response rate could not be calculated.

Surveys were administered through Qualtrics. No financial incentives were offered. Incomplete survey responses were excluded, and the analytic sample comprised all 633 complete eligible responses obtained during the recruitment period. State workforce estimates were used to contextualize the sample but were not treated as a probability sampling frame [23]. The analytic dataset used in the present study is distinct from the dataset used in the 2025 North Dakota nursing‐retention work conducted by the North Dakota Nursing Research Collaborative [24].

### 2.2. Measures

The survey collected demographic variables (age, gender identity, and educational degree) and employment variables (professional role, direct patient‐care status, years in healthcare, rural or urban workplace designation, and work setting). These variables were summarized descriptively; the primary inferential analysis focused on the eight focal study constructs specified by the study objectives.

ProQOL: The 30‐item ProQOL‐5 comprises three 10‐item subscales—compassion satisfaction, burnout, and STS. Items are rated on a 5‐point scale from 1 (*never*) to 5 (*very often*), with higher subscale scores indicating more of the corresponding construct [6, 25]. In this study, Cronbach’s *α* was 0.90 for compassion satisfaction, 0.80 for burnout, and 0.83 for STS.

Organizational commitment: Meyer and Allen’s model assess affective, continuance, and normative commitment [9, 26]. Affective commitment reflects emotional attachment to the organization, continuance commitment reflects perceived costs of leaving, and normative commitment reflects perceived obligation to remain. Higher subscale scores indicate stronger commitment to the corresponding dimension. Cronbach’s *α* values in this study were 0.93, 0.88, and 0.89, respectively.

Team cohesion: The 13‐item Erlangen Team Cohesion at Work Scale comprises a six‐item collegial solidarity subscale and a seven‐item unity/problem management subscale and uses a five‐point agreement response format; higher subscale scores indicate greater team cohesion [14]. Cronbach’s *α* values were 0.91 for collegial solidarity and 0.87 for unity/problem management.

Intent to leave: Intent to leave was measured with a four‐item, 5‐point Likert scale assessing attitudes toward leaving the current employer [4]. Item scores were summed (possible range, 4–20), with higher scores indicating stronger intent to leave. Cronbach’s *α* was 0.86.

### 2.3. Data Analysis

Analyses were conducted using SAS Version 9.4. Means and standard deviations summarized continuous variables, and frequencies and percentages summarized categorical variables. Pearson correlations assessed bivariate relationships. The primary multiple linear regression simultaneously entered the three ProQOL subscales, three organizational commitment dimensions, and two team‐cohesion subscales. Demographic and employment variables were not included as covariates in the primary model because the inferential objective focused on the eight study constructs. Linearity was evaluated using scatterplots, multicollinearity using variance inflation factors (all < 5), and homoscedasticity using residual plots. The primary regression used 633 complete cases; no imputation was performed. Statistical significance was set at *p* < 0.05. Because the STS coefficient differed in direction from its bivariate association with intent to leave, post hoc nested regressions were conducted to determine when the coefficient changed direction. Models sequentially entered STS alone burnout, compassion satisfaction, the three organizational commitment dimensions, and the two team‐cohesion subscales. Robustness was examined using HC3 heteroskedasticity‐robust standard errors, influence screening, and a sensitivity model adjusting for age, professional role, direct patient‐care status, years in healthcare, rural/urban workplace designation, and work setting. OpenAI ChatGPT (GPT‐5.6 Sol; accessed August 21, 2026) was used after completion of the original manuscript as a computational aid during the revision process. A deidentified participant‐level analytic dataset was provided in the ChatGPT workspace to support the development and checking of Python code for post hoc analyses; no direct identifiers were included. All model specifications and reported results were independently checked against the primary SAS outputs. A complete coefficient table for the sensitivity model adjusting for age, professional role, direct patient‐care status, years in healthcare, rural/urban workplace designation, and work setting is provided in Supporting Table S1.

## 3. Results

Descriptive statistics for study variables are presented in Table 1. The final sample included 633 participants; 608 (96.1%) were women, 386 (61.0%) worked in rural settings, and 508 of 632 participants with valid responses (80.4%) reported direct patient‐care roles. Registered nurses were the largest professional group (*n* = 433, 68.4%). Hospitals were the most common work setting (*n* = 252, 39.8%), followed by clinics (*n* = 180, 28.4%) and nursing homes (*n* = 163, 25.8%). Demographic and employment characteristics are summarized in Table 2.

**TABLE 1:** Descriptive statistics for study variables.

Variable	*M*	SD
Secondary traumatic stress	20.50	6.07
Burnout	22.50	5.90
Compassion satisfaction	39.99	5.85
Organizational commitment, total	4.32	1.03
Affective commitment	4.59	1.41
Continuance commitment	4.23	1.44
Normative commitment	4.13	1.23
Team cohesion, total	3.71	0.75
Collegial solidarity	3.85	0.79
Unity/problem management	3.59	0.76
Intent to leave	9.95	4.33

*Note: N* = 633, ProQOL = Professional Quality of Life.

Abbreviation: STS = secondary traumatic stress.

**TABLE 2:** Demographic and employment characteristics (*N* = 633).

Characteristic	Category	*n*	%
Age	18–24 years	26	4.1
	25–40 years	254	40.1
	41–56 years	232	36.7
	57–64 years	93	14.7
	65–75 years	26	4.1
	76 + years	2	0.3

Education	High school/equivalent	75	11.9
	Associate degree	172	27.2
	Baccalaureate degree	280	44.2
	Master’s degree	79	12.5
	Doctoral degree	27	4.3

Professional role	Registered nurse	433	68.4
	Certified nursing assistant	74	11.7
	Licensed practical/vocational nurse	55	8.7
	Nurse practitioner	41	6.5
	Advanced practice registered nurse	9	1.4
	Certified registered nurse anesthetist	2	0.3
	Other	19	3.0

Years in healthcare	0–4	52	8.2
	5–10	89	14.1
	11–20	205	32.4
	21–30	160	25.3
	≥ 31	127	20.1

Work setting	Hospital	252	39.8
	Clinic	180	28.4
	Nursing home	163	25.8
	Other	38	6.0

Gender	Woman	608	96.1
	Man	25	3.9

Direct patient care	Yes	508	80.4
	No	124	19.6

Workplace designation	Rural	386	61.0
	Urban	247	39.0

*Note:* “Other” indicates respondents who selected, “Other, not listed” on the professional role item. The analytic dataset does not contain a write‐in professional‐role field; therefore, the specific roles in this category could not be further characterized. Direct patient‐care percentages are based on 632 valid responses; one response was missing.

Bivariate correlations (Table 3) showed that intent to leave was positively correlated with STS (*r* = 0.16) and burnout (*r* = 0.49) and negatively correlated with compassion satisfaction (*r* = −0.42), affective commitment (*r* = −0.62), continuance commitment (*r* = −0.19), normative commitment (*r* = −0.38), collegial solidarity (*r* = −0.33), and unity/problem management (*r* = −0.36). STS and burnout were positively correlated (*r* = 0.63).

**TABLE 3:** Pearson correlations among study variables.

Variable	1	2	3	4	5	6	7	8	9	10
1. Secondary traumatic stress	—									
2. Burnout	0.63^∗∗^	—								
3. Compassion satisfaction	−0.29^∗∗^	−0.65^∗∗^	—							
4. Affective commitment	−0.09^∗^	−0.42^∗∗^	0.47^∗∗^	—						
5. Continuance commitment	0.13^∗∗^	0.09^∗^	0.01	0.24^∗∗^	—					
6. Normative commitment	−0.02	−0.19^∗∗^	0.26^∗∗^	0.51^∗∗^	0.35^∗∗^	—				
7. Team cohesion, total	−0.18^∗∗^	−0.41^∗∗^	0.37^∗∗^	0.51^∗∗^	0.01	0.14^∗∗^	—			
8. Collegial solidarity	−0.17^∗∗^	−0.41^∗∗^	0.36^∗∗^	0.47^∗∗^	0.02	0.12^∗∗^	0.95^∗∗^	—		
9. Unity/problem management	−0.18^∗∗^	−0.38^∗∗^	0.35^∗∗^	0.50^∗∗^	0.01	0.15^∗∗^	0.96^∗∗^	0.84^∗∗^	—	
10. Intent to leave	0.16^∗∗^	0.49^∗∗^	−0.42^∗∗^	−0.62^∗∗^	−0.19^∗∗^	−0.38^∗∗^	−0.36^∗∗^	−0.33^∗∗^	−0.36^∗∗^	—

*Note: N* = 633. Values are Pearson’s *r*.

^∗^ *p* < 0.05.

^∗∗^ *p* < 0.01.

The primary multiple regression (Table 4) identified four variables independently associated with intent to leave after simultaneous adjustment and explained 47% of the variance in intent to leave, *R* ^2^ = 0.47, *F* (8, 624) = 68.64, *p* < 0.001. Burnout was positively associated with intent to leave after adjustment (*β* = 0.40, *p* < 0.001), whereas affective commitment (*β* = −0.41, *p* < 0.001) and continuance commitment (*β* = −0.10, *p* = 0.003) were inversely associated. STS also showed a statistically significant negative adjusted association (*β* = −0.11, *p* = 0.005), despite its positive bivariate correlation with intent to leave. Compassion satisfaction, normative commitment, collegial solidarity, and unity/problem management were not independently associated with intent to leave after adjustment for the other model variables.

**TABLE 4:** Multiple regression model for intent to leave.

Variable	*B*	95% CI	*β*	*t*	*p*
Intercept	12.22	[8.64, 15.80]	—	6.71	< 0.001
Secondary traumatic stress	−0.08	[−0.13, −0.02]	−0.11	−2.80	0.005
Burnout	0.29	[0.22, 0.37]	0.40	7.74	< 0.001
Compassion satisfaction	0.02	[−0.04, 0.08]	0.02	0.58	0.561
Affective commitment	−1.25	[−1.50, −0.99]	−0.41	−9.57	< 0.001
Continuance commitment	−0.29	[−0.48, −0.10]	−0.10	−2.98	0.003
Normative commitment	−0.24	[−0.49, 0.01]	−0.07	−1.92	0.056
Collegial solidarity	0.41	[−0.18, 1.01]	0.08	1.36	0.173
Unity/problem management	−0.45	[−1.07, 0.17]	−0.08	−1.43	0.153

*Note:* Complete‐case *n* = 633. *R* ^2^ = .47; *F* (8, 624) = 68.64; *p* < 0.001. *B* = unstandardized coefficient; *β* = standardized coefficient.

Abbreviation: CI = confidence interval.

### 3.1. Post Hoc Analysis of the STS Coefficient Reversal

Nested regression showed that the STS coefficient changed direction specifically when burnout entered the model (Table 5). With STS alone, STS was positively associated with intent to leave (*B* = 0.116, *β* = 0.163, *p* < 0.001; *R* ^2^ = 0.027). After burnout was added, STS became negative (*B* = −0.175, *β* = −0.246, *p* < 0.001; *R* ^2^ = 0.277). The negative STS coefficient persisted as compassion satisfaction, organizational commitment, and team cohesion were added, reaching *B* = −0.079, *β* = −0.111, *p* = 0.005 in the full model. This pattern is consistent with a suppression or coefficient‐reversal effect associated with shared variance between STS and burnout.

**TABLE 5:** Nested regression models examining the STS coefficient reversal.

Model	Variables entered	*R* ^2^	STS *B*	STS *β*	STS *p*
1	STS	0.027	0.116	0.163	< 0.001
2	+ Burnout	0.277	−0.175	−0.246	< 0.001
3	+ Compassion satisfaction	0.285	−0.157	−0.220	< 0.001
4	+ Affective, continuance, and normative commitment	0.466	−0.075	−0.105	0.008
5	+ Collegial solidarity and unity/problem management	0.468	−0.079	−0.111	0.005

*Note. N* = 633. Models were entered sequentially. *B* = unstandardized coefficient; *β* = standardized coefficient. Model 5 is the prespecified primary eight‐construct model.

Abbreviation: STS = secondary traumatic stress.

The STS result was robust to alternative specifications. With HC3 robust standard errors, STS remained significant (*p* = 0.006). The coefficient also remained negative and significant after influence screening and after adjustment for selected demographic and employment covariates (*B* = −0.083, *p* = 0.004; *n* = 632); the covariate block added 2.5% explained variance and was not statistically significant as a block (Δ*R* ^2^ = 0.025, *p* = 0.075).

## 4. Discussion

The findings indicate that occupational strain and organizational attachment are central correlates of nursing personnel’s intent to leave. Burnout had the largest positive adjusted association (*β* = 0.40), while affective commitment had the largest negative standardized coefficient (*β* = −0.41), consistent with prior research linking these constructs to turnover‐related outcomes [8, 11, 27].

The present findings are broadly consistent with recent studies linking professional quality‐of‐life domains with turnover‐related outcomes [17–21]. The added contribution of this analysis is the simultaneous estimation of occupational well‐being, relational/team cohesion, and multidimensional organizational commitment in a statewide nursing workforce, allowing the independent adjusted associations of these correlated constructs—and the STS coefficient reversal after burnout adjustment—to be evaluated within a single model.

Affective commitment was the largest inverse adjusted association. Continuance commitment also remained significant after adjustment, although its effect was smaller. Normative commitment was inversely related to intent to leave at the bivariate level but did not reach statistical significance in the multivariable model (*β* = −0.07, *p* = 0.056). The adjusted findings therefore support a more specific interpretation: Emotional attachment to the organization and, to a lesser extent, perceived costs of leaving were independently associated with lower intent to leave, whereas a perceived obligation to remain did not contribute unique explanatory value after the other predictors were considered.

### 4.1. Burnout and Secondary Traumatic Stress

Burnout had the largest positive adjusted association with intent to leave (*β* = 0.40). Chronic exhaustion and adverse responses to sustained workplace demands can diminish emotional and physical resources and make continued employment feel less sustainable. The present finding is consistent with prior evidence identifying burnout as an important antecedent of turnover intention and reinforces the need to treat burnout primarily as an organizational workforce issue rather than solely as an individual coping problem [8, 27]. For nursing management, the result places workload, staffing adequacy, recovery opportunities, and responsive leadership near the center of the retention strategy.

STS showed a more complex pattern. It was positively correlated with intent to leave at the bivariate level (*r* = 0.16), but nested regression demonstrated that its coefficient became negative, specifically when burnout entered the model. STS and burnout were substantially correlated (*r* = 0.63). This observed coefficient reversal is consistent with a suppression pattern in which adjustment for correlated burnout partitions shared variance and changes the association represented by the residual component of STS [28]. The negative adjusted coefficient should not be interpreted as evidence that greater STS protects against intent to leave; rather, it describes the association of STS with intent to leave conditional on burnout and the other modeled predictors. The persistence of the negative coefficient across robust and demographic sensitivity analyses strengthens confidence that the reversal is not attributable to a small number of influential observations or the measured demographic characteristics.

### 4.2. Organizational Commitment and Intent to Leave

Affective commitment was negatively associated with intent to leave and had the largest standardized negative coefficient in the final model. Nursing personnel who identify with organizational values, experience belonging, and feel emotionally connected to their employer may be more inclined to remain even when work is demanding. This pattern is consistent with Meyer and Allen’s three‐component model and nursing research demonstrating inverse relationships between affective commitment and turnover intention. Because affective commitment reflects a desire to remain rather than a perceived necessity to do so, it is a particularly important target for leadership‐ and culture‐focused retention efforts [9, 11].

Continuance commitment was also negatively associated with intent to leave, although its effect was smaller. This dimension reflects awareness of the costs associated with leaving, including accumulated benefits, seniority, professional relationships, or limited alternative employment opportunities. Continuance commitment may stabilize employment, but it does not necessarily indicate enthusiasm for the organization. Leaders should therefore avoid relying on barriers to departure as a primary retention mechanism and instead pair competitive employment conditions with practices that build engagement, professional growth, and genuine organizational attachment [10].

Normative commitment showed a moderate negative bivariate association with intent to leave (*r* = −0.38), but it was not statistically significant after simultaneous adjustment for professional quality of life, the other commitment dimensions, and team cohesion (*β* = −0.07, *p* = 0.056). This attenuation suggests that normative commitment shares explanatory variance with other organizational and individual factors in the model. The result should therefore be interpreted as evidence of a bivariate relationship, not as an independent adjusted association [9, 12].

### 4.3. Compassion Satisfaction and Team Cohesion

Compassion satisfaction was associated with intent to leave at the bivariate level but did not remain significant after burnout, STS, and organizational commitment were considered simultaneously. This pattern may indicate that the protective value of professional fulfillment overlaps with other dimensions of well‐being and attachment. Similarly, the team‐cohesion subscales did not explain unique variance in the final regression despite their conceptual relevance. These findings should not be taken to mean that collegial relationships are unimportant; rather, their effects may be indirect, shared with organizational commitment, or more evident for outcomes such as engagement, job satisfaction, and team performance [15, 16, 25].

### 4.4. Implications for Nursing Management

The adjusted associations identify burnout and affective commitment as priority domains for nursing‐management action and prospective testing rather than as interventions proven to reduce turnover. Healthcare organizations could evaluate strategies such as workload and staffing review, access to psychological support, structured recovery opportunities, flexible scheduling, relational leadership, meaningful recognition, psychological safety, shared governance, and professional development. Their effects on intent to leave and actual turnover should be assessed prospectively [29–33].

Management strategies could also examine whether practices that foster belonging and perceived value of remaining are associated with stronger affective and continuance commitment. Recognition, supportive leadership, professional development, equitable employment policies, and scheduling flexibility are plausible candidates for local implementation and evaluation. Because this cross‐sectional study cannot establish causality, these strategies should be regarded as evidence‐informed priorities for testing rather than interventions demonstrated by the present data to reduce turnover. [34–36].

### 4.5. Strengths and Limitations

The strengths of this study include a comparatively large statewide sample spanning multiple nursing roles and settings, validated measures, simultaneous consideration of constructs spanning occupational/individual, relational/team, and organizational domains, and post hoc analyses that localized the STS coefficient reversal to adjustment for burnout. The cross‐sectional design precludes causal inference. Convenience sampling and recruitment within one state limit generalizability, and open electronic recruitment prevented calculation of a conventional response rate. Because all study measures were self‐reported, common‐method and response biases are possible. Demographic and employment characteristics were not included in the prespecified primary regression, although sensitivity adjustment did not materially alter the principal coefficients. The nested STS analyses were post hoc and therefore require replication; they identify a statistical suppression pattern but cannot establish the causal mechanism underlying shared variance between burnout and STS. Unmeasured mediators or moderators may further influence the observed relationships [37, 38].

### 4.6. Recommendations for Further Research

Subsequent research should move beyond cross‐sectional association testing toward longitudinal and intervention‐based designs that clarify temporal ordering and evaluate whether changes in workplace conditions produce changes in turnover intention. Studies should test multicomponent interventions addressing workload, burnout, trauma‐related stress, leadership, recognition, and organizational commitment rather than evaluating these factors in isolation. Such designs could help identify which combinations of strategies have the greatest practical value for retention [37, 38].

The STS coefficient reversal remains a priority for replication and mechanistic investigation. The present nested analyses showed that the reversal occurred when burnout entered the model, but future studies should use preregistered longitudinal or structural equation approaches to clarify how trauma‐related stress and burnout develop over time and to distinguish suppression, mediation, and moderation mechanisms. Replication in other geographic regions and nursing workforces is needed before the adjusted negative STS coefficient is generalized. Additional mediation and moderation analyses should also examine why compassion satisfaction and team cohesion did not explain unique variance. Rural–urban comparisons and analyses by professional role, years of experience, and practice setting may identify contexts in which these relationships differ [19].

## 5. Conclusion

Burnout, affective commitment, continuance commitment, and STS were independently associated with intent to leave in the multivariable model in this cross‐sectional North Dakota sample. Burnout had the largest positive adjusted association, whereas affective commitment had the largest negative standardized coefficient. Nested regression showed that the STS coefficient changed from positive to negative when burnout entered the model, supporting a coefficient‐reversal/suppression interpretation arising from shared variance; the negative adjusted coefficient should not be interpreted as a protective effect. Normative commitment, compassion satisfaction, and team cohesion did not explain significant unique variance after adjustment. These findings identify occupational strain and organizational attachment as candidates for integrated retention‐strategy testing. Longitudinal and intervention‐based research should determine whether leadership and workforce strategies targeting these domains produce sustained reductions in intent to leave or actual turnover.

## Funding

This research received no external funding.

## Ethics Statement

The study received approval from the University of North Dakota Institutional Review Board (Protocol No. IRB0006279) and was conducted in accordance with applicable U.S. ethical requirements for research involving human participants. Participants provided informed consent before participation and were informed that participation was voluntary, responses were anonymous, and withdrawal was permitted at any time before survey submission.

## Conflicts of Interest

The authors declare no conflicts of interest.

## Supporting Information

Additional supporting information can be found online in the Supporting Information section.

## Supporting information


**Supporting Information 1** Supporting Table S1 presents the complete coefficient table for the sensitivity model adjusted for age, professional role, direct patient‐care status, years in healthcare, rural or urban workplace designation, and work setting. It provides additional detail supporting the robustness analysis reported in the Results section.

**Supporting Information 2** STROBE cross‐sectional reporting checklist.

## Data Availability

The deidentified data supporting the findings of this study are available from the corresponding author upon reasonable request, subject to applicable ethical and institutional requirements.
