# Supplementary material for: Professional Quality of Life, Team Cohesion, Organizational Commitment, and Intent to Leave Among Nursing Personnel: A Cross‐Sectional Study
Source: J Nurs Manag. 2026 Sep 30;2026:9984390. doi: 10.1155/jonm/9984390 (PMC13625668; doi:10.1155/jonm/9984390)
Supplement: Supplementary file 1 [file JONM-2026-9984390-s002.docx]

**STROBE Cross-Sectional Reporting Checklist**

*Manuscript: Professional Quality of Life, Team Cohesion, Organizational Commitment, and Intent to Leave Among Nursing Personnel: A Cross-Sectional Study*

| **Item** | **STROBE topic** | **Recommendation (summary)** | **Where/how addressed** | **Location** |
| --- | --- | --- | --- | --- |
| 1 | Title and abstract | Identify the observational design and provide an informative, balanced abstract. | Cross-sectional design appears in the title; structured abstract summarizes rationale, methods, key results, conclusion, and management implications. | p. 1 |
| 2 | Background/rationale | Explain the scientific background and rationale. | Introduction explains turnover burden and rationale for examining professional quality of life, team cohesion, and organizational commitment together. | pp. 2–3 |
| 3 | Objectives | State specific objectives and any prespecified hypotheses. | Three study objectives are stated explicitly. No directional hypotheses were prespecified. | pp. 3–4 |
| 4 | Study design | Present key elements of the study design early. | Methods identifies a cross-sectional electronic survey and the analytic approach. | p. 4 |
| 5 | Setting | Describe setting, locations, and relevant dates. | North Dakota healthcare settings; recruitment and data collection occurred October 2024 through March 2025. | pp. 4–5 |
| 6 | Participants | Give eligibility criteria and sources/methods of participant selection. | Eligibility (age ≥18 and employment in a North Dakota healthcare setting), convenience sampling, recruitment channels, and complete-response inclusion are described. | p. 4 |
| 7 | Variables | Define outcomes, predictors, potential confounders/effect modifiers, and diagnostic criteria if relevant. | Intent to leave is the outcome; eight focal scale predictors and demographic/employment variables are defined. The primary model focused on the eight focal constructs. | pp. 5–6 |
| 8 | Data sources/measurement | For each variable, describe data sources and measurement methods; address comparability where relevant. | Validated instruments, item/subscale structure, response formats, scoring interpretation, and internal consistency are reported. | pp. 5–6 |
| 9 | Bias | Describe efforts to address potential sources of bias. | Open recruitment and inability to calculate a conventional response rate are reported; validated measures were used; convenience sampling, self-report, common-method, and response biases are acknowledged as limitations. | pp. 4, 12–13 |
| 10 | Study size | Explain how the study size was determined. | The analytic sample comprised all 633 complete eligible responses obtained during the recruitment period; no probability sampling frame was used. | pp. 4–5 |
| 11 | Quantitative variables | Explain how quantitative variables were handled and, if grouped, how groupings were chosen. | Scale scoring and direction are described; continuous focal scale scores were analyzed without categorization. Categorical demographic groupings are shown in Table 2. | pp. 5–6; Table 2, pp. 23–24 |
| 12a | Statistical methods | Describe statistical methods, including approaches to control for confounding. | Descriptive statistics, Pearson correlations, simultaneous multiple regression, diagnostic checks, and post hoc nested/sensitivity analyses are described. | pp. 6–7 |
| 12b | Subgroups/interactions | Describe methods for subgroup or interaction analyses. | No prespecified subgroup analysis was part of the primary model. A post hoc demographic/employment sensitivity model is reported; interaction analyses are not presented as primary findings. | pp. 6–8 |
| 12c | Missing data | Explain how missing data were addressed. | Primary regression used 633 complete cases with no imputation. One direct-patient-care response was missing; the demographic sensitivity model used n = 632. | pp. 6–8; Table 2, p. 24 |
| 12d | Sampling strategy | If applicable, describe methods accounting for the sampling strategy. | Convenience/open electronic recruitment is stated. No sampling weights or probability-sampling adjustments were used. | p. 4 |
| 12e | Sensitivity analyses | Describe sensitivity analyses. | HC3 robust standard errors, influence screening, and demographic/employment covariate adjustment were used to evaluate robustness of the STS coefficient reversal. | pp. 6–8 |
| 13a | Participants—numbers | Report numbers at relevant study stages. | 633 complete eligible responses formed the analytic sample; descriptive counts are provided. | pp. 4, 7; Table 2, pp. 23–24 |
| 13b | Non-participation | Give reasons for non-participation at each stage when available. | The invitation denominator could not be established because recruitment used open electronic dissemination; therefore, a conventional response rate and reasons for non-participation could not be calculated. | p. 4 |
| 13c | Flow diagram | Consider a participant flow diagram. | Not provided because this was a single-stage anonymous electronic survey without a known invitation denominator; participant accounting is described in text. | p. 4 |
| 14a | Descriptive data | Report participant characteristics and relevant exposures/confounders. | Age, education, role, tenure, setting, gender, direct patient care, and rural/urban designation are reported. | p. 7; Table 2, pp. 23–24 |
| 14b | Missing data—descriptive | Indicate missing data for variables of interest. | Table 2 notes one missing direct-patient-care response; focal scale variables used in the primary model were complete for all 633 cases. | pp. 6–7; Table 2, p. 24 |
| 14c | Follow-up time | Summarize follow-up time when relevant. | Not applicable to a cross-sectional design. | Not applicable |
| 15 | Outcome data | Report numbers or summary measures for outcome variables. | Intent-to-leave mean and SD are reported in Table 1; bivariate associations and multivariable results are reported in Tables 3–5. | Table 1, p. 22; Tables 3–5, pp. 25–26 |
| 16a | Main results | Provide unadjusted and adjusted estimates with precision; identify confounders adjusted for and why. | Zero-order correlations are in Table 3; the prespecified eight-predictor regression reports B, 95% CI, β, t, and p. The Methods explains why demographics were excluded from the primary model and reports sensitivity adjustment. | pp. 6–8; Tables 3–4, pp. 25–26 |
| 16b | Category boundaries | Report category boundaries when continuous variables are categorized. | Focal predictors and outcome were analyzed as continuous scores; no analytic categorization was used. | pp. 5–6 |
| 16c | Relative-risk translation | When relevant, translate relative risk to an absolute risk for a meaningful period. | Not applicable; the outcome is a continuous intent-to-leave score analyzed with linear regression. | Not applicable |
| 17 | Other analyses | Report other analyses, including sensitivity analyses. | Post hoc nested regression localized the STS coefficient reversal to the addition of burnout; robust and demographic sensitivity analyses are reported. | p. 8; Table 5, p. 26 |
| 18 | Key results | Summarize key results with reference to study objectives. | Discussion opens by summarizing burnout, affective commitment, continuance commitment, and the STS coefficient-reversal pattern. | pp. 9–10 |
| 19 | Limitations | Discuss limitations, including direction/magnitude of possible bias or imprecision. | Cross-sectional design, convenience sampling, generalizability, self-report/common-method bias, covariate specification, and post hoc nature of nested analyses are addressed. | pp. 12–13 |
| 20 | Interpretation | Provide a cautious overall interpretation considering objectives, limitations, multiplicity, and other evidence. | Discussion interprets results cautiously, explicitly rejects a protective interpretation of the adjusted STS coefficient, and situates findings in prior research. | pp. 9–14 |
| 21 | Generalisability | Discuss external validity/generalizability. | Single-state convenience sampling is identified as limiting generalizability; replication across regions and nursing workforces is recommended. | pp. 13–14 |
| 22 | Funding | State funding source and role of funders. | The study received no external funding. | p. 15; title page |

This author-completed checklist maps the manuscript to the STROBE recommendations for cross-sectional observational studies. Item wording is summarized for submission convenience. Source: https://www.strobe-statement.org/checklists/

Reference: von Elm, E., Altman, D. G., Egger, M., Pocock, S. J., Gøtzsche, P. C., & Vandenbroucke, J. P. (2007). The Strengthening the Reporting of Observational Studies in Epidemiology (STROBE) statement: Guidelines for reporting observational studies. PLoS Medicine, 4(10), e296. https://doi.org/10.1371/journal.pmed.0040296
